# An uncommon and easily overlooked case: Delayed intraperitoneal bladder rupture following blunt trauma: A case report and review of the literature

**DOI:** 10.1097/MD.0000000000037147

**Published:** 2023-02-02

**Authors:** Man Ma, Gongbin Wei, Chaopu Liu, Yanan Xu

**Affiliations:** a Department of Traumatology, Chongqing Emergency Medical Center/Chongqing University Central Hospital, Chongqing, China.

**Keywords:** bladder wall hematoma, case report, CT cystography, delayed intraperitoneal bladder rupture, microscopic hematuria

## Abstract

**Introduction:**

Delayed intraperitoneal bladder rupture is a rare clinical occurrence, frequently overlooked and misdiagnosed due to its nonspecific clinical manifestations. However, literature provides only a limited number of cases reporting delayed intraperitoneal bladder rupture resulting from blunt abdominal injury.

**Patient concerns:**

A 72-year-old female pedestrian was struck by a vehicle and experienced sudden, severe abdominal pain on the 8th day following the injury. Abdominal B-ultrasound revealed a significant accumulation of peritoneal effusion. The abdominal puncture retrieved serosanguinous ascites. Then the patient was promptly transferred to our hospital. Upon transfer, the physical examination revealed the patient vital signs to be stable, accompanied by mild abdominal distension, slight tenderness, tension, and an absence of rebound tenderness. Urinalysis detected microscopic hematuria, while contrast-enhanced computed tomography (CT) revealed considerable fluid accumulation in the abdominal cavity, without evidence of solid organ damage, and the bladder was adequately filled.

**Diagnosis:**

The diagnosis of delayed intraperitoneal bladder rupture primarily relied on intraoperative observations.

**Interventions:**

An emergency exploratory laparotomy was performed, revealing a linear rupture at the dome of the bladder. Subsequently, the bladder rupture was repaired.

**Outcomes:**

Postoperative cystography demonstrated full recovery and the patient was discharged 28 days post-surgery. The postoperative recovery was uneventful without any complications.

**Conclusions:**

A well-distended bladder observed in CT does not definitively rule out the potential for bladder injury. False negatives may occur due to incomplete bladder filling during CT cystography. Retrograde cystography can identify cases missed by CT cystography. In cases of substantial intra-abdominal free fluid, surgical intervention should be actively considered for patients with blunt abdominal trauma without concurrent solid organ damage.

## 1. Introduction

Traumatic bladder injuries are rare, comprising only 0.78% to 1.6% of blunt abdominal injuries, with 85% of them being blunt injuries.^[1]^ The rarity of bladder injuries is primarily attributed to the protective pelvic structure, typically stemming from high-energy trauma. Clinical signs of traumatic bladder injuries are nonspecific and mainly involve gross hematuria, suprapubic pain/tenderness, and urinary difficulties.^[2,3]^ Clinicians can easily diagnose and treat bladder injuries when typical clinical signs are present, but patients with atypical symptoms, especially delayed bladder rupture, may be overlooked or misdiagnosed, potentially impacting treatment efficacy. In this study, we present a rare and easily overlooked case of delayed intraperitoneal bladder rupture resulting from blunt trauma. Ethical approval for this study was obtained from the ethics committee of the Chongqing Emergency Medical Center. The patient and her family were informed about emergency exploratory laparotomy procedure and relevant medical documents were signed after obtaining their consent.

## 2. Case presentation

**Figure 1.**
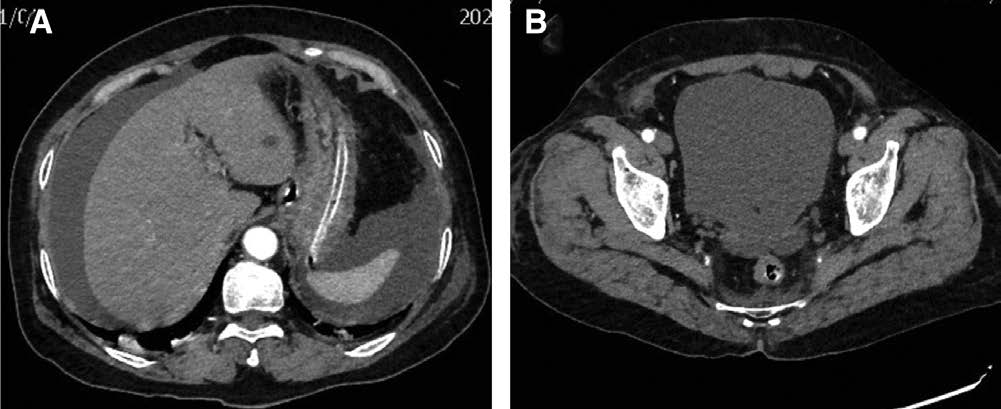
**Preoperative abdominal contrast-enhanced CT scan.** (A) Substantial volume of free fluid was present in the perihepatic and splenic fossae. (B) The bladder was adequately filled. CT = computed tomography.

**Figure 2.**
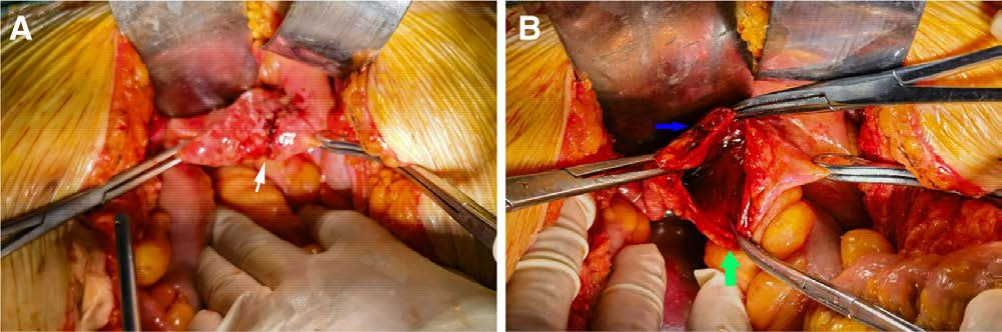
**Bladder injury detected during surgery.** (A) Linear contusion measuring 3 cm in length was identified in the bladder dome (white arrow). (B) A full-thickness bladder rupture was evident (green arrow), accompanied by a hematoma within the bladder wall (blue arrow).

A 72-year-old female pedestrian, who had previously undergone hysterectomy and adnexectomy due to cervical cancer, was struck by a vehicle. She complained of headache, dizziness, right hip and right knee pain with limited mobility. She was taken to the local hospital 1 hour after the injury. Computed tomography (CT) examination revealed a complex pattern of injuries including right frontotemporal, parietal, and occipital subdural hematoma, scalp hematoma, right superior and inferior pubic ramus and acetabulum fractures, and right tibial plateau fracture. Consequently, she was admitted to the neurosurgery department. The patient could urinate independently after the injury, and routine urine analysis showed no obvious abnormalities. Repeated abdominal CT examination did not show significant abdominal fluid. On the 8th day post-injury, the patient experienced sudden severe abdominal pain. Abdominal CT revealed the accumulation of abdominal fluid, prompting immediate treatment with gastrointestinal decompression and catheterization. The catheterization procedure proceeded smoothly, with clear yellow urine. The abdominal puncture retrieved ascitic fluid like meat washing water. Due to the deterioration of the patient condition, she was promptly transferred to our hospital. Upon arrival, she was fully conscious and her vital sign was stable. The abdominal examination showed mild distension, slight tenderness, and tension, with no rebound pain. Contrast-enhanced CT reexamination revealed substantial abdominal fluid accumulation, and the bladder was fully distended with no evidence of solid organ injuries (Fig. 1). CT cystography likewise indicated the absence of bladder injury, while urinalysis showed microscopic hematuria. An emergency exploratory laparotomy was conducted, revealing approximately 2000 mL of clear yellow liquid in the abdominal cavity. A 3 cm linear contusion was identified on the bladder dome (Fig. 2A). There was no hematoma or any blood in the peritoneal cavity. The intraoperative bladder injection test was positive, the bladder wall was easily separated from the full thickness, and hematoma was found in the bladder wall adjacent to the laceration (Fig. 2B). During the surgery, urine ascites was drained, and the bladder laceration was repaired. Subsequent postoperative cystography confirmed complete recovery, and the patient was discharged 28 days after the surgery with an uneventful recovery and no complications.

## 3. Discussion

Extraperitoneal bladder ruptures are predominantly linked to pelvic fractures, constituting around 80% of major bladder ruptures, while intraperitoneal ruptures account for about 15%.^[4]^ Clinical symptoms of traumatic bladder injury lack specificity. Over 95% of patients with bladder rupture exhibit gross hematuria, while 5% display only microscopic hematuria.^[5]^ In this case, CT confirmed the presence of a pelvic fracture, yet only microscopic hematuria was observed after the injury, and no significant traumatic bladder changes were evident in multiple abdominal CT scans. Mucosal lacerations and bladder wall contusions are unlikely to appear as significant abnormalities on CT or cystography, even when clinical signs like hematuria are present.^[2]^ According to the American Urological Association Core Trauma Guidelines, a pelvic fracture with only microscopic hematuria carries a <1% risk of concomitant bladder injury.^[6]^ It is evident that subtle or occult bladder rupture can be easily missed when they coincide with pelvic fractures and only manifest as microscopic hematuria.

Conventional retrograde cystography and CT cystography are commonly employed methods for clinical bladder injury detection.^[2]^ Both methods were known for their high sensitivity and specificity in diagnosing bladder injuries, including intraperitoneal and extraperitoneal injuries.^[7]^ Given that pelvic fractures and intraperitoneal bladder injuries are frequently associated with high-energy trauma, CT scans are often necessary to promptly assess for visceral injury and bleeding in such patients. Therefore, to save times, CT cystography is routinely employed in clinical practice to assess the presence of concurrent bladder injury.^[2]^ Delayed imaging enhances the chances of detecting bladder rupture, but the diagnostic accuracy of such antegrade contrast studies may be compromised by inadequate bladder distension.^[8]^ In this case, the preoperative CT scan detected free intraperitoneal fluid but revealed no traumatic changes in the bladder. The primary reason for the false negative result was incomplete bladder filling, preventing the contrast medium from overflowing. Conventional retrograde cystography involves instilling approximately 350 mL of contrast medium via a Foley catheter, ensuring full bladder distension and reducing the risk of missed diagnoses. Studies have reported that this approach achieves a sensitivity of 92.8% in detecting extraperitoneal bladder rupture, and 100% sensitivity in detecting intraperitoneal rupture.^[9]^ In this case, the presence of intraperitoneal bladder rupture was additionally confirmed through an intraoperative bladder injection test, reaffirming the utility of retrograde cystography in identifying false negative cases of bladder injury overlooked by CT cystography.

On the 8th day post-injury, the patient experienced abrupt and severe abdominal pain. Emergency laparotomy showed no active bleeding in the bladder dome. The edges of the bladder rupture appeared macerated, suggesting that the lesion had been present for several days. This presented as a classic case of delayed bladder rupture. It has been reported that delayed presentation of intraperitoneal bladder rupture following trauma may result from the masking of a primary laceration or the development of secondary rupture at the site of a hematoma or another lesion in the bladder wall.^[10]^ During surgery blood clots were discovered in the bladder wall, suggesting the formation of a hematoma in the bladder wall after the injury. This aligns with the literature reports mentioned earlier, confirming that delayed bladder rupture was a secondary rupture induced by hematoma in the bladder wall. During surgery, it was observed that there was merely a linear crack at the top of the bladder, without any apparent laceration or urine leakage. Nonetheless, during the bladder injection test, significant urine leakage was detected at the crack, suggesting that the crack would open as the bladder reached a certain level of filling. Weyrauch and Peterfy also conducted experimental studies in dogs, demonstrating that small tears in the bladder may spontaneously seal without leaking until a certain level of bladder distension is reached.^[10]^ This provides a further explanation for why the bladder could still fill adequately after urine had spilled into the abdominal cavity.

The majority of extraperitoneal bladder ruptures (approximately 85%) spontaneously close within 10 days, and nearly all close within 3 weeks. If catheterization does not effectively drain the bladder, conservative therapy is not suitable.^[11]^ All intraperitoneal bladder ruptures resulting from blunt abdominal trauma required formal repair. They tend to be larger than what cystography indicates and are unlikely to heal through conservative management. This situation can lead to severe, and occasionally fatal complications, especially sepsis.^[12]^ Indications necessitating immediate surgery in patients with blunt abdominal trauma include the presence of isolated free fluid without solid organ injuries, particularly in cases of abdominal pain or following a car accident.^[13]^ In this case, although the preoperative CT did not detect any bladder injury and only identified isolated free intraperitoneal fluid without solid organ injury, an emergency laparotomy was conducted, ultimately confirming the rupture of the bladder dome during the procedure. Excluding solid organ injuries in blunt abdominal trauma, the typical causes of intra-abdominal fluid collection on abdominal CT include a perforated bowel, mesentery injury and intraperitoneal bladder rupture.^[13]^ Despite the low incidence of bladder injury in patients with pelvic fractures and microscopic hematuria, awareness of the potential for minor or occult intraperitoneal bladder injuries is crucial, as delayed bladder rupture may occur in such cases. The limitation of this case lies in the blind emergency laparotomy conducted without confirming the existence of bladder rupture through retrograde cystography prior to surgery. While the patient eventually had a positive outcome, performing endoscopic bladder repair could have been a better option if the presence of intraperitoneal bladder rupture had been confirmed by retrograde cystography before surgery. Such an approach is less traumatic for the patient and promotes a quicker recovery.

## 4. Conclusions

A well-distended bladder observed in CT does not definitively rule out the potential for bladder injury. False negatives may occur due to incomplete bladder filling during CT cystography. Retrograde cystography can identify cases missed by CT cystography. In cases of substantial intra-abdominal free fluid, especially when accompanied by abdominal pain or following a vehicular accident, surgical intervention should be actively considered for patients with blunt abdominal trauma without concurrent solid organ damage.

## Acknowledgments

This report is published with the consent of the patient. We are very grateful to the patient for her support and trust in our hospital.

## Author contribution

**Methodology:** Gongbin Wei, Chaopu Liu.

**Project administration:** Gongbin Wei, Chaopu Liu, Yanan Xu.

**Supervision:** Chaopu Liu.

**Writing – original draft:** Man Ma.

**Writing – review & editing:** Yanan Xu.
